# The missing link between legal age of sexual consent and age of marriage in sub-Saharan Africa: implications for sexual and reproductive health and rights

**DOI:** 10.1186/s12978-021-01177-w

**Published:** 2021-06-21

**Authors:** Bright Opoku Ahinkorah, Joshua Okyere, John Elvis Hagan, Abdul-Aziz Seidu, Richard Gyan Aboagye, Sanni Yaya

**Affiliations:** 1 School of Public Health, Faculty of Health, University of Technology Sydney, Ultimo, NSW Australia; 2 Department of Population and Health, College of Humanities and Legal Studies, University of Cape Coast, Cape coast, Ghana; 3 Department of Health Promotion, Education and Disability Studies, Kwame Nkrumah University of Science and Technology, Kumasi, Ghana; 4 Neurocognition and Action-Biomechanics-Research Group, Faculty of Psychology and Sport Sciences, Bielefeld University, Bielefeld, Germany; 5 College of Public Health, Medical and Veterinary Sciences, James Cook University, Townsville, Queensland Australia; 6 Takoradi Technical University, P.O. Box 257, Takoradi, Ghana; 7 School of Public Health, University of Health and Allied Sciences, Ho, Ghana; 8 School of International Development and Global Studies, University of Ottawa, Ottawa, Canada; 9 The George Institute for Global Health, Imperial College London, London, United Kingdom

**Keywords:** Adolescents, Age of marriage, Sexual consent, Sub-Saharan Africa

## Abstract

Child marriage is a fundamental violation of human rights and a threat to access to education, sexual and reproductive health care, and employment. It also threatens freedom from violence, reproductive rights, movement, and the right to consensual marriage. In most countries in sub-Saharan Africa, the legal age of marriage is 18 years. Hence, girls who marry before 18 years are considered as victims of child marriage. Closely knitted to legal age for marriage is the issue of age for sexual consent, which refers to the minimum age at which a person is considered to have the legal capacity to consent to sexual intercourse. While there seem to be a standard legal age for marriage, the legal age for sexual consent varies in most countries in sub-Saharan Africa and is often lower than the legal age of marriage. In this commentary, we argue that the gap between the legal age of sexual consent and marriage partly accounts for some of the sexual and reproductive health challenges such as intimate partner violence, sexually transmitted infections, adolescent pregnancy, early childbirth, including unsafe abortions among adolescent girls in sub-Saharan Africa and infringements on their sexual and reproductive health rights. This commentary highlights strategic potential interventions that could help address the identified gaps. We argue that aligning the age for sexual consent and marriage is not the solution to the problem. However, what is critical is the education of young people about sexual and reproductive health issues and comprehensive sexuality education through advocacy networks at the national and local levels. Thus, the key is to provide accurate, timely, and non-judgmental sexual and reproductive health and rights information to young people irrespective of the prevailing age of consent. This provision will empower them to make informed decisions about their sexual and reproductive health.

## Background

Ensuring sexual and reproductive health and rights (SRHR) of adolescents are encapsulated in human rights instruments and policies that have been signed, ratified, and adopted by individual states and nations [1]. This is against the backdrop that improving adolescents’ SRHR is fundamental to the advancement and guarantee of a crises-free adolescence and productive life later as adults [1, 2]. Therefore, in situations where adolescents are denied this right because of policy absence or inconsistencies, it becomes an important social and public health concern that ought to be addressed with expediency. When adolescents are denied of SRHR, it predisposes them to a plethora of risky sexual behaviours, social delinquencies and adverse sexual and reproductive health (SRH) outcomes such as adolescent pregnancies, sexually transmitted infections (STIs), including HIV among others [1, 3].

Internationally, there is consensus that child marriage is an infringement of the rights of a child, disproportionately affecting girls worldwide [4–6]. Among the international agreements that have led the discourse on age for marriage is the Universal Declaration of Human Rights; Convention on Consent to Marriage, Minimum Age for Marriage, and Registration of Marriages; Convention on the Elimination of All Forms of Discrimination against Women (CEDAW); and the Convention on the Rights of the Child (CRC) [7]. As such, the United Nations Department of Economic and Social Affairs [8] has made a call for the elimination of child marriage by the year 2030. The question lingers on what constitutes child marriage? According to the United Nations convention on the rights of the child, child marriage encapsulates any marriage wherein both partners or one partner is below the age of 18, with or without consent [9]. Parsons et al. [10] also suggest that child marriage involves any legal or customary union involving a boy or a girl before age 18. Thus, child marriage can be a formal or informal union; it can be legal or customary; it involves marriage before age 18; and can be forced or voluntary.

It is estimated that, 600 million girls marry before age 18 globally [8]. In low-and middle-income countries, one in three girls marry before age 18 whereas one in five girls marry before age 18 in high income countries [4]. Although the practice of girl child marriage has markedly declined over the past two decades, it remains pervasive in many societies across the world, with sub-Sahara Africa accounting for approximately 50–70% of girls being married before age 18 years [5, 6, 11]. The emphasis on girls in this context is based on the premise that girls are more likely to marry before age 18 compared to their male counterparts [12].

Surprisingly, although the legal age for marriage is set at 18 for most populations, it remains a grey area in some jurisdiction. For instance, many regions (e.g., Africa) lack an explicitly defined minimum age for marriage when the minors’ parents’ consent to the union [13–15]. Nonetheless, across sub-Saharan Africa, the prevalence of child marriage is highest in West Africa (49%), followed by Central Africa (40%). Within Central Africa, the Democratic Republic of Congo dominates with 74% of all girls in sexual unions by age 19. Cameroon follows with 52% of girls 20–24 years married by age 18. The highest rates of women aged 20–24 who were first married or in sexual unions by age 15 are found in Niger, Chad, and Mali across West Africa [16].

**Fig. 1:**
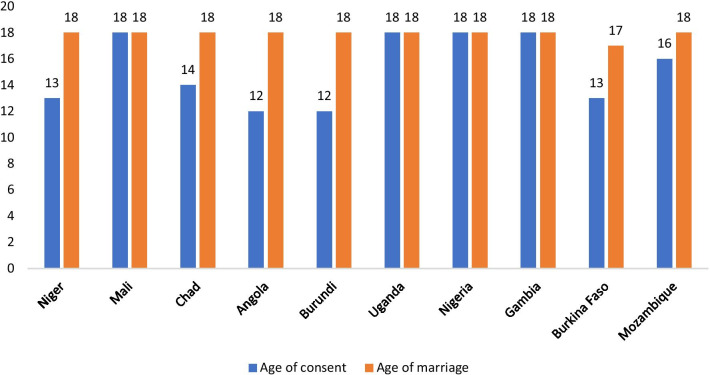
Legal age of consent and the fertility rates of the ten sub-Saharan African countries with fertility rates above 5.0

**Fig. 2:**
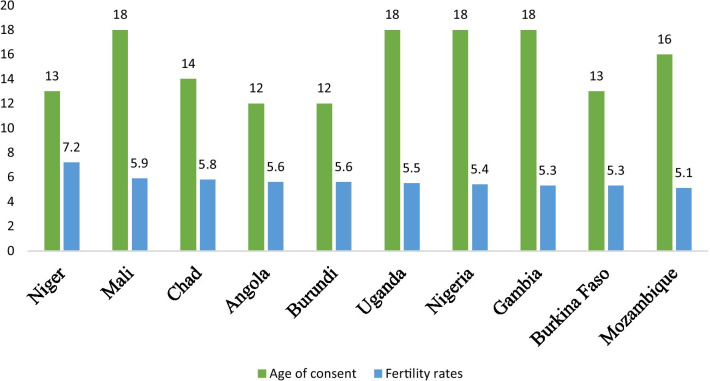
Legal age of consent and the fertility rates of the ten sub-Saharan African countries with fertility rates above 5.0

Closely knitted to legal age for marriage is the issue of age for sexual consent. Age for sexual consent refers to the minimum age at which a person is considered to have the legal capacity to consent to sexual intercourse [17]. Unlike marriage, there is not a universally recognised standard for the age of sexual consent. As such, the age for sexual consent varies substantially across the globe. For instance, in Angola and Burundi, the age for sexual consent stands at 12 years whereas in Bahrain, it stands at 21 years [17]. Fig. 1 shows the legal age of consent and marriage in ten sub-Saharan African countries with fertility rates above 5.0. As shown in Fig. 1, except for girls in Mali, Uganda, and Nigeria, the legal age of sexual consent is lower in the ten countries in sub-Saharan Africa with fertility rates above 5.0. For some of the countries (e.g., Angola, Burundi, and Niger), the difference between the legal age of consent and marriage is between 5 and 6 years. The question is, what happens in between the legal age of consent and marriage? The low age consent may explain the high fertility rate in most countries in sub-Saharan Africa. Fig. 2 shows the legal age of consent and the fertility rates of the ten sub-Saharan African countries with fertility rates above 5.0.

Meanwhile, in some jurisdiction, there is no legal age for sexual consent. This trend is because traditionally, many societies link age for sexual consent to the timing of puberty [18]. However, given that the age for puberty has decreased in recent times, it raises concerns about age for sexual consent and its implications on the SRH of young people. Therefore, this review discusses the missing link between legal age for marriage and age for sexual consent and its associated implications, using Ghana as a case study.

### International frameworks on sexual and reproductive health and rights

Respect and recognition of SRHR in the world can be traced back to the Programme of Action that was accepted at the 1994 International Conference on Population and Development held in Cairo, Egypt. The Programme of Action defined reproductive health and identified its key tenets which includes “family planning, maternal health care, safe abortion when not against the law, education on sexuality and reproductive health, and prevention and appropriate treatment of infertility, reproductive tract infections, and sexually transmitted diseases” [19]. Later in 1995, the Beijing Fourth World Conference of Women (FWCW) reaffirmed the significance of SRHR through its Platform for Action [20]. At the FWCW, it was affirmed that ‘The human rights of women include their right to have control over and decide freely and responsibly on matters related to their sexuality including, SRH, free of coercion, discrimination and violence’ [21]. SRHR is further emphasised in the CEDAW and the Convention on the Rights of Persons with Disabilities [22].

The past decades have seen two international agreements targeted at protecting the rights of children in sub-Saharan Africa: the 1989 United Nations CRC and the 1990 African Charter on the Rights and Welfare of the Child (ACRWC) [23]. Till date, all African countries have sanctioned or signed up with the CRC except Somalia where several child rights are seriously violated through child marriage. Another complementary follow-up of ACRWC reiterated the need for all signatories to implement strategic actions to end child marriage by adopting a policy to set the minimum age for marriage at 18 and ensure that all marriages are compulsorily registered [24].

Beyond these, there was also the Millennium Development Goals (MDGs) of which goal 5b focused on attaining universal access to reproductive health [25]. However, the targets of the MDGs were not fully attained, hence, ushering the world into the Sustainable Development Goals (SDGs). Currently, the SDG 3, target 3.7 calls for nations to ensure universal access to SRHR [26]. All these policies and frameworks have shaped and continue to shape the development of SRHR across the globe and in Africa.

### What is the situation in sub-Saharan Africa: the Ghanaian Case

Unless otherwise stated, specific issues peculiar to Ghana are highlighted. Ghana like other countries in sub-Saharan Africa place high premium on the SRH needs and concerns of young people. As such, the country has set the legal age for marriage, and the age for sexual consent as a means of protecting young people from sexual abuse, and repercussions of engaging in early marriages or early sexual initiation. Legally, the Criminal Offences Act, 1960 (Act 29) specifies the age for sexual consent to be 16 years [27]. On the other hand, the legal age for marriage as enshrined in the Children’s Act 1998 (Act 560) Section 14(2) is at 18 years [28]. This mismatch implies that, there is a two-year gap between the legal age for sexual consent and age for  marriage in Ghana. So, it is legal to have sex at age 16, but criminal to marry at age 16 or before age 18? This paradox in the legal and regulatory framework on age for marriage and sexual consent presents a social and legal conundrum. More so, this disparity in the legal age for marriage vis-a-vis age for sexual consent has raised concerns about increasing the age for sexual consent to be at par with the legal age for marriage in Ghana. However, we should be concerned about what happens in between the gap period as captured in the two legal frameworks.

### What happens in between?

The issues that happen within the gap between age for sexual consent and the legal age for marriage in Ghana can be viewed from a pluralist perspective. On one hand, it has tremendous positive outcomes on the SRH of young people. On the other hand, it may pose as a problem for SRH. From an optimist point of view, the setting of the age for sexual consent relatively lower than age for marriage provides an avenue for young people to have access to SRH information and services in order to make informed decisions [17]. Within the Ghanaian context, sexual communication is lacking and shrouded in secrecy as the agencies for socialization (i.e., parents, the family, school, church, and community) refrain from open discussions on sexuality matters. According to Amo-Adjei [29], the usual concern is about the appropriate age to begin orienting young people about SRH issues and the contents that should be divulged. Meanwhile, the age for sexual consent could have been used as a proxy to guide when to start SRH education and socialization. Granted that the framers of legal age for sexual consent framework at 16 years provided an opportunity for young people to access accurate SRH information that would guide them in their sexual decision making between that period and the point of marriage, the potential negative implications were somewhat overlooked.

Several problems are likely to be experienced as a result of the disparity between age for sexual consent and the legal age for marriage. In Ghana, the relatively younger age for sexual consent in comparison to age for marriage is perceived as an endorsement of early sexual initiation [17]. Admittedly, the prevailing socio-cultural norms and beliefs abhor premarital sex [30]. As such, younger people are socialised to believe that they must wait till marriage or the legal age for marriage before they can consent to sex. It is important to note that not only is age 18 the legal age for marriage in Ghana; it also doubles as the legal age of adulthood [31]. Therefore, the legal provision that positions the age for sexual consent at 16 years seemingly creates an enabling environment for young people to engage in sexual activities earlier than expected by socio-cultural norms. Hence, the tendency for sexual activity to occur between the window age for sexual consent and the legal age for marriage is relatively high.

Also, this gap between the legal age sexual consent and marriage exposes young people, particularly girls to the risk of intimate partner violence (IPV). From a legal and cultural position, a young person is considered a minor at 16 years. In most cases, the sexual relation happens between the child and an adult. Therefore, older sexual partners are likely to take advantage of their younger counterparts. Moreover, sexual negotiation is very low at age 16. As such, young people are unable to negotiate for safe sex [32].

Given the disparity between the age for sexual consent and age for marriage, young people may engage in sexual experimentation. It is worthy of note that age 16 is within the period of adolescence where the individual is exploring his/her sexuality [33]. Therefore, this gap in between age for sexual consent and age for marriage provides the perfect premise to justify engagement in sexual activities and sexual experimentation which otherwise would have not sufficed if age for sexual consent and legal age for marriage were aligned. At age 16, the rationale for engaging in sexual activities is not for procreation but for fun or out of curiosity. This sexual experimentation can have short-term and long-term effects on the individual such as contraction of STI’s, including HIV and AIDs.

Another critical issue is the perpetuation of child marriage due to the mismatch sexual windows. As stated previously, sexual negotiation at age 16 is very unlikely to happen. As such, young girls in particular are coerced to engage in unprotected sex which result in teenage pregnancy. Therefore, to prevent shame and disgrace, parents force their children to marry or live with the person who got them pregnant [34]. This phenomenon is very common in the coastal communities in Ghana where girls who get pregnant are forced to live with the expectant father and his family [35].

### What are the impacts on sexual and reproductive health and rights?

The disparity between age for sexual consent and age for marriage comes with a host of problems which are associated with several health effects. Due to the issue of sexual experimentation and inability to insist on protected sex at age 16, young people are at high risk of contracting STIs such as HIV, syphilis, gonorrhoea, and human papilloma virus (HPV) [36]. Girls who are unable to bear the shame and stigma associated with getting pregnant at that age resort to unsafe abortion because most of them are not privy to accurate SRH information. This act sometimes leads to adverse health complications, disability and death [36]. Nevertheless, the prevalence of unsafe abortions and abortion-related complications and mortalities would have been far worse if the age for sexual consent and age for marriage were to be aligned. This is because, in such circumstances, young people cannot seek abortion care since health professionals who provide such services would be practicing illegally [17]. However, at 16 years, the biological constitute of the girl may pose as a threat to childbirth. Girls who engage in sexual activities are more likely to get pregnant and when they do, they are at  risk of experiencing stillbirths [37], miscarriages [38], and having low birth weight children [39] due to young maternal age at birth.

Not only does the mismatch between age for sexual consent and age for marriage result in deleterious health consequences, it also impacts significantly on the human rights of adolescents. Both the 1994 ICPD and the 1995 Beijing Platform for Action recognise access to SRH information and services as a right for the adolescent [20]. However, this right is infringed in situations where there is confusion in relation to the laws and policies that regulate the age for sexual consent and age for marriage. For instance, in Zimbabwe, it is reported that confusion related to the legal frameworks on age of consent exacerbates the stigma about adolescent sexuality and subsequently leads to the denial of access to SRH information and services to adolescents who need it most [40].

### What is the way forward: “righting the mismatch between laws and policies”

Observing from afar, one might be tempted to propose that the age for sexual consent and marriage should be aligned as a conduit to resolve the problems and health consequences created by the current legal provision on age for sexual consent and marriage. However, we posit that the status quo should remain. This assumption is because laws regarding age of marriage and age of sexual consent are imperfect interventions to resolve the issues [17]. For instance, in Ghana, like many sub-Saharan countries (e.g., Nigeria, Benin, Togo), numerous confounding determinants may sway the appropriateness of its laws. Legal frameworks would also battle against context-specific sociocultural norms (e.g., familial, and ethnic endorsement) of early marriages, especially among girls.

Although some of these countries offer criminalization, others ban or annul marriages if one of the partners is younger than the legally approved minimum age whereas other countries covertly support the act customarily [23]. Essentially, increasing the age for consent without close-in-age exemptions can be problematic. What happens is that, increasing the age for consent legitimizes the criminalization of sexual activities among adolescents. This approach would eventually limit adolescents’ access to SRH information and services, and that could retard the attainment of SDG target 3.7 that seeks to ensure universal access to sexual and reproductive health care services, including family planning services, information, and education by 2030.

Therefore, attention and priority should be channelled towards the education of young people about SRH issues and comprehensive sexuality education through advocacy networks at the national and local levels. This approach will empower young people and help them to make informed decisions about their SRH. Thus, the key is to provide accurate, timely, and non-judgmental SRHR information to adolescents irrespective of the prevailing age of consent. When accurate information and comprehensive sexuality education [29] are made available to adolescents, they would appreciate that having age for sexual consent at 16 years does not imply that they can engage in risky sexual behaviour like multiple sexual partnership, sexual experimentation, and unsafe sex. Through comprehensive sexuality education, young people who engage in sexual activity at 16 years and get pregnant would be aware that they can access comprehensive abortion care to reduce the risk of complications, disability, and death [41]. Without accurate information and comprehensive sexuality education, we may even set the age for sexual consent at 21 years and still encounter these identified problems.

It is important for sub-Saharan Africa to roll out pathways to ensure equitable access to SRH services to sexually active adolescents. According to Petroni, Das, and Sawyer [17], sexuality is an intrinsic component of humans and is increased with the onset of puberty. Given that the age for puberty has decreased significantly across the globe [17], it implies that many young people are sexually active. Therefore, providing equitable access to SRH services such as family planning and comprehensive abortion care would be quintessential to resolving the problems associated with the current status of Africa’s legal age for marriage and sexual consent. Since diverse family, societal, cultural, and religious influences build unfriendly environment for interactions on adolescent sexuality matters in many parts of sub-Saharan Africa, SRH programme organizers should target specific interventions that focus on continuous awareness creation, attitudinal change, and personal skills (e.g., decision making) to help address the specific needs of adolescents. Providing adolescent user-friendly sexual and reproductive health services devoid of pre-judgmental or stereotypical behaviours against adolescents who would want to seek for accurate SRH information is essential [42, 43].

## Conclusion

The disparity between the legal age for sexual consent and age for marriage has raised concerns about what happens between the gap. This review provides an insight into the nuances surrounding having an age for sexual consent that is relatively lower than the age for marriage. We conclude that this disparity has a pluralistic outlook, i.e., having both positive and negative outcomes on the young person. However, we posit that aligning both the age for marriage and age for sexual consent would not make a significant difference. Instead, sub-Sahara Africa should focus on instituting comprehensive sexuality education in schools and also for out-of-school young people, as well as improve access to SRH services through the provision of adolescent-and-youth-friendly corners and centres, family planning and comprehensive abortion care. These strategies will ensure that sub-Sahara Africa addresses the problems associated with the current status quo of the age for marriage and sexual consent. Future empirical studies should investigate whether girls and the general population are aware of the minimum age of marriage and sexual consent laws and their effectiveness through nation-wide surveys or examine the trends over time using longitudinal designs.

## Data Availability

Not applicable.

## Abbreviations

CRC: Convention on the Rights of the Child
CEDAW: Convention on the Elimination of All Forms of Discrimination against Women
HIV: Human Immunodeficiency Virus
SRHR: Sexual and reproductive health and rights
STIs: Sexually transmitted infections

## References

[1] Tallarico R, Ozah K, Orievulu KS (2021). Age of consent: a case for harmonizing laws and policies to advance, promote and protect adolescents’ sexual and reproductive health rights. African J Reprod Health..

[2] Santhya KG, Jejeebhoy SJ (2015). Sexual and reproductive health and rights of adolescent girls: evidence from low-and middle-income countries. Global public health..

[3] Galati AJ. Onward to 2030: sexual and reproductive health and rights in the context of the Sustainable Development Goals.

[4] de Groot R, Kuunyem MY, Palermo T (2018). Child marriage and associated outcomes in northern Ghana: a cross-sectional study. BMC Public Health..

[5] UNICEF. Progress for children: a World fit for children: statistical review. 2007. http://www.unicef.org/progressforchildren/2007n6/index_41848.htm. Accessed 3 May 2021.

[6] Unicef. The state of the world’s children: maternal and newborn health. New York. 2009. http://www.unicef.org/sowc09/docs/ SOWC09-FullReport-EN.pdf. Accessed 3 May 2021.

[7] Mathur S, Greene M, Malhotra A. Too young to wed: the lives, rights and health of young married girls.

[8] United Nations Department of Economic and Social Affairs. The Sustainable Development Goals Report 2018. New York, NY. 2018. https://unstats.un.org/sdgs/files/report/2018/TheSustainableDevelopmentGoalsReport2018-EN.pdf.

[9] Assembly UG (1989). Convention on the rights of the child. United Nations, Treaty Series..

[10] Parsons J, Edmeades J, Kes A, Petroni S, Sexton M, Wodon Q (2015). Economic impacts of child marriage: a review of the literature. Rev Faith Int Affairs..

[11] Black M. Early Marriage: Child Spouses. Innocenti Digest No. 7. http://www.unicef-icdc.org/publications/pdf/digest7e.pdf. Accessed 3 May 2021.

[12] Koski A, Heymann J (2018). Child marriage in the United States: how common is the practice, and which children are at greatest risk?. Perspect Sexual Reprod Health..

[13] Center TJ. Falling through the cracks: How laws allow child marriage to happen in today’s America. http://www.tahirih.org/wp-content/uploads/2017/08/TahirihChildMarriageReport-1.pdfm. Accessed 25 May 2021.

[14] United Nations. The Convention on the Rights of the Child. 2015. https://treaties.un.org/Pages/ViewDetails.aspx?mtdsg_no=IV11&chapter=4&lang=en. Accessed on 3 May 2021.

[15] UNICEF. The African Charter on the Rights and Welfare of the Child, 2015. http://www.unicef.org/esaro/children_youth_5930.html. Accessed 3 May 2021.

[16] Walker JA (2012). Early marriage in Africa–trends, harmful effects and interventions. Afr J Reprod Health..

[17] Petroni S, Das M, Sawyer SM (2019). Protection versus rights: age of marriage versus age of sexual consent. Lancet Child Adolescent Health..

[18] Bullough VL (2005). Age of consent: a historical overview. J Psychol Hum Sexuality.

[19] Starrs AM, Ezeh AC, Barker G, Basu A, Bertrand JT, Blum R, Coll-Seck AM, Grover A, Laski L, Roa M, Sathar ZA (2018). Accelerate progress—sexual and reproductive health and rights for all: report of the Guttmacher-Lancet Commission. Lancet..

[20] Burke JP, Coles SM, Meglio DG, Gibson JE, Handschin MS, Lau M, Marcell VA, Tebb PK, Urbach K (2014). Sexual and reproductive health care: a position paper of the Society for Adolescent Health and Medicine. J Adolesc Health..

[21] World Conference on Women (4th: 1995: Beijing, China). Report of the Fourth World Conference on Women: Beijing, 4–15 September 1995. [New York]: United Nations; 1996.

[22] Della Fina V, Cera R, Palmisano G, editors. The United Nations convention on the rights of persons with disabilities: A commentary. Springer; 2017.

[23] Maswikwa B, Richter L, Kaufman J, Nandi A (2015). Minimum marriage age laws and the prevalence of child marriage and adolescent birth: evidence from sub-Saharan Africa. Int Perspect Sexual Reprod Health..

[24] Awuye VE. A Study of the Implementation of International Treaties on Child Marriage in Ghana (Doctoral dissertation, University of Ghana).

[25] Temmerman M, Khosla R, Say L (2014). Sexual and reproductive health and rights: a global development, health, and human rights priority. Lancet..

[26] Sciortino R (2020). Sexual and reproductive health and rights for all in Southeast Asia: more than SDGs aspirations. Cult Health Sexuality..

[27] Act 29 of Ghana. Criminal offences Act, 1960.

[28] Abane A, Acheampong FO, Adjaloo MK, Ampong GO, Clerk G, Hampshire K, Kilpatrick K, Porter G, Twum-Danso A. Children's rights in Ghana: reality or rhetoric? Lexington Books; 2011.

[29] Amo-Adjei J. Toward an understanding of optimal grade for starting sexuality education programme for in-school children and adolescents: insights from ghana. Am J Sexuality Edu. 2021; 1–9.

[30] Anarfi JK, Owusu AY (2011). The making of a sexual being in Ghana: the state, religion and the influence of society as agents of sexual socialization. Sexuality Cult..

[31] Mahama S, Tackie-Ofosu V, Nyarko NY (2018). Conceptions of adulthood: perspectives from Ghana. IFE PsychologIA Int J.

[32] van der Riet M, Sofika D, Akhurst J, Daniels H (2019). Young people’s investments in sexual relationships: a different prioritization of self in the negotiation of safe sex practices in South Africa. Sexualities..

[33] Blakemore SJ, Mills KL (2014). Is adolescence a sensitive period for sociocultural processing?. Ann Rev Psychol.

[34] Efevbera Y, Bhabha J (2020). Defining and deconstructing girl child marriage and applications to global public health. BMC public health..

[35] Okyere J. Defining Child Marriage in Ghana. Modern Ghana. 2020 https://www.modernghana.com/news/1026073/defining-child-marriage-in-ghana.html.

[36] Santhya KG, Ram U, Acharya R, Jejeebhoy SJ, Ram F, Singh A (2010). Associations between early marriage and young women's marital and reproductive health outcomes: evidence from India. Int Perspect Sexual Reprod Health..

[37] Wilson RE, Alio AP, Kirby RS, Salihu HM (2008). Young maternal age and risk of intrapartum stillbirth. Arch Gynecol Obstetrics..

[38] Asamoah B, Kjellstrom T, Östergren PO (2018). Is ambient heat exposure levels associated with miscarriage or stillbirths in hot regions? A cross-sectional study using survey data from the Ghana Maternal Health Survey 2007. Int J Biometeorol.

[39] Dennis JA, Mollborn S (2013). Young maternal age and low birth weight risk: an exploration of racial/ethnic disparities in the birth outcomes of mothers in the United States. Social Sci J.

[40] Amnesty International. Lost without knowledge: barriers to sexual and reproductive health information in Zimbabwe. 2018. https://www.amnesty.org/download/Documents/AFR4677002018ENGLISH.PDF. Accessed 4 June 2021.

[41] Amo-Adjei J, Darteh EK (2017). Unmet/met need for contraception and self-reported abortion in Ghana. Sexual Reprod Healthcare..

[42] Ahinkorah BO, Hagan JE, Seidu A-A, Budu E, Hormenu T, Mintah JK, Sambah F, Schack T (2019). Access to adolescent pregnancy prevention information and services in Ghana: a community-based case-control study. Front Public Health.

[43] Ahinkorah BO, Hagan JE, Seidu A-A, Mintah JK, Sambah F, Schack T, Hormenu T (2019). Examining pregnancy related socio-cultural factors among adolescent girls in the komenda-edina-eguafo-abrem municipality in the Central Region of Ghana: a case-control study. Front Public Health.

