# Correction to: The missing link between legal age of sexual consent and age of marriage in sub-Saharan Africa: implications for sexual and reproductive health and rights

**DOI:** 10.1186/s12978-021-01190-z

**Published:** 2021-07-01

**Authors:** Bright Opoku Ahinkorah, Joshua Okyere, John Elvis Hagan, Abdul-Aziz Seidu, Richard Gyan Aboagye, Sanni Yaya

**Affiliations:** 1 School of Public Health, Faculty of Health, University of Technology Sydney, Ultimo, NSW Australia; 2 Department of Population and Health, College of Humanities and Legal Studies, University of Cape Coast, Cape Coast, Ghana; 3 Department of Health Promotion, Education and Disability Studies, Kwame Nkrumah University of Science and Technology, Kumasi, Ghana; 4 Neuro-Cognition and Action-Biomechanics-Research Group, Faculty of Psychology and Sport Sciences, Bielefeld University, Bielefeld, Germany; 5 College of Public Health, Medical and Veterinary Sciences, James Cook University, Townsville, QLD Australia; 6 Takoradi Technical University, P.O. Box 257, Takoradi, Ghana; 7 School of Public Health, University of Health and Allied Sciences, Ho, Ghana; 8 School of International Development and Global Studies, University of Ottawa, Ottawa, Canada; 9 The George Institute for Global Health, Imperial College London, London, UK

## Correction to: Reprod Health (2021) 18:128 https://doi.org/10.1186/s12978-021-01177-w

In the original publication of this article [1] the captions of figures 1 and 2 were identical. In this correction article the correct captions are published. The original article has been updated (Figs. 1, 2).

**Fig. 1:**
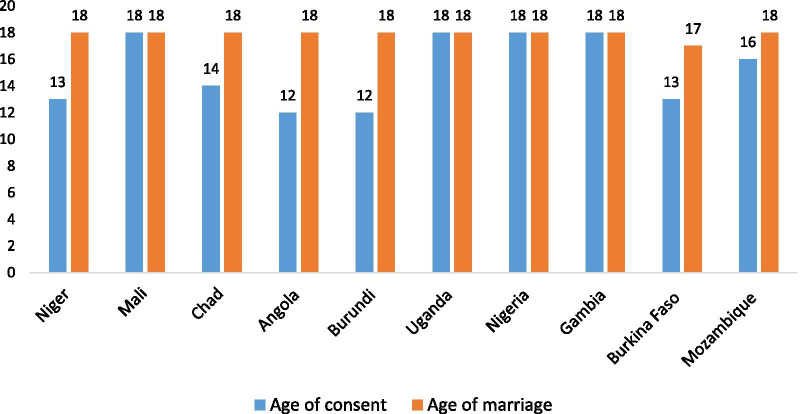
Legal age of consent and the age of marriage in the ten sub-Saharan African countries with fertility rates above 5.0

**Fig. 2:**
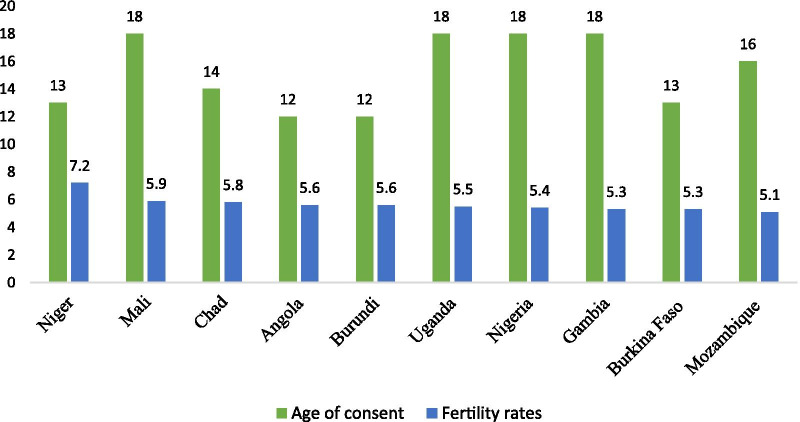
Legal age of consent and the fertility rates of the ten sub-Saharan African countries with fertility rates above 5.0
